# The birth of modern sports nutrition: tracing the path from muscle biopsies to creatine supplementation—A narrative review

**DOI:** 10.1080/15502783.2025.2463373

**Published:** 2025-02-18

**Authors:** Jeffrey R. Stout, Richard B. Kreider, Darren G. Candow, Scott C. Forbes, Eric S. Rawson, Brandi Antonio, Jose Antonio

**Affiliations:** a University of Central Florida, School of Kinesiology and Rehabilitation Sciences, Orlando, Florida, USA; b Texas A&M University, Department of Kinesiology and Sports Management, College Station, TX, USA; c University of Regina, Faculty of Kinesiology and Health Studies, Regina, SK, Canada; d Brandon University, Department of Physical Education Studies, Brandon, MB, Canada; e Messiah University, Department of Health, Nutrition, and Exercise Science, Mechanicsburg, PA, USA; f Nova Southeastern University, Department of Health and Human Performance, Davie, FL, USA

**Keywords:** Sports nutrition, muscle metabolism, exercise physiology, ergogenic aids, exercise performance

## Abstract

**Background:**

Modern sports nutrition has evolved through discoveries in muscle metabolism and dietary supplementation. Advances in muscle biopsy techniques revealed how diet influences muscle energetics and exercise performance. The establishment of the Metabolic Research Laboratory provided a platform for further investigation, leading to the identification of creatine monohydrate (CrM) as an effective ergogenic aid. This review outlines the historical development of sports nutrition research from the 1960s to the early 1990s, highlighting key breakthroughs in muscle glycogen metabolism, dietary interventions, and creatine supplementation.

**Methods:**

We conducted a narrative review that combined personal accounts with seminal research studies. This approach allowed us to examine the contributions of Drs. Jonas Bergström and Eric Hultman—founders of the Metabolic Research Laboratory—as well as the early work of their postdoctoral colleague, Dr. Roger Harris.

**Results:**

Muscle biopsy techniques enabled direct analysis of muscle metabolism, leading to insights into glycogen depletion and recovery. The Metabolic Research Laboratory advanced our understanding of muscle energetics and informed dietary strategies for enhancing performance. In 1992, the rediscovery of CrM supplementation demonstrated its capacity to increase intramuscular creatine levels, significantly improving exercise performance and recovery. These breakthroughs reshaped sports nutrition and expanded its relevance to clinical and aging populations.

**Conclusion:**

The progression from early muscle metabolism research to the validation of CrM supplementation underscores how foundational laboratory discoveries have shaped modern sports nutrition. The work of the Metabolic Research Laboratory and its key investigators continues to inform applications in both performance enhancement and clinical health.

## 1. Introduction

Since the 1960s, sports nutrition has undergone transformative advances that have revolutionized our understanding of athletic performance, muscle metabolism, and exercise physiology (Table 1). This narrative review features firsthand perspectives from pioneering researcher Dr. Roger Harris, tracing the evolution from Dr. Jonas Bergström’s groundbreaking muscle biopsy technique development to the discovery of creatine monohydrate (CrM) supplementation efficacy.

**Table 1.** Pivotal moments shaping the future.

Year	Event
1959	Development of Blood Glucose Assay – Hultman published his method in *Nature* titled, “Rapid, specific method for determination of aldosaccharides in body fluids.”
1962	Development of Muscle Biopsy Technique – Bergström’s modified muscle biopsy technique revolutionized exercise physiology by enabling direct examination of muscle metabolism before and after exertion, advancing our understanding of fatigue mechanisms.
1966	Discovery of Muscle Glycogen’s Role in Exercise – Jonas Bergström and Eric Hultman published a groundbreaking study that quantified human muscle glycogen levels for the first time and established its relationship with exercise and diet. This study fundamentally changed the understanding of how diet affects exercise performance.
1967	Influence of Diet on Exercise Performance – A study by Jonas Bergström, Bengt Saltin, and L. Hermansen demonstrated that muscle glycogen levels, influenced by diet, directly affected exercise endurance. This research was the first to show the direct impact of diet on exercise performance.
1974	High-Energy Phosphates and Exercise Study – Roger Harris published a highly influential paper on ATP, PCr, and total creatine levels in human muscles at rest.
1975	Initial Creatine Supplementation Study (unpublished) – A preliminary study by Bergström, Hultman, Harris, and Sahlin attempted to increase muscle creatine levels through supplementation in two swimmers but incorrectly concluded that muscle creatine levels would not increase through diet.
1983	Harris’s Transition to Equine Research – Roger Harris transitioned to researching muscle metabolism in horses, which eventually led to new insights and circled back to impact human sports nutrition, particularly in developing creatine supplementation.
1992	Breakthrough in Creatine Supplementation – Roger Harris discovered that oral creatine supplementation could significantly increase muscle creatine content and enhance performance. This breakthrough in sports nutrition sparked widespread interest in ergogenic aids.
1992	Real-World Impact on Athletic Performance – A newspaper article spotlighted the real-world impact of Harris and Hultman’s research on creatine supplementation. Their scientific findings were put into practice by British athletes during the Barcelona Olympics, demonstrating the practical application of creatine in sports nutrition.

Beginning with Dr. Bergström and Dr. Eric Hultman’s collaborations and the development of the Metabolic Research Laboratory at the Karolinska Institute and St. Eriks Hospital in Sweden, which laid the foundation for modern sports science by demonstrating the first direct links between diet and exercise performance [1]. This review chronicles the progression of research through the Metabolic Research Laboratory, highlighting notable contributions from the first post-docs under Drs. Bergström and Hultman, including Drs. Norman McLennan-Anderson, Kent Sahlin, and Roger Harris, whose work on muscle energetics played a pivotal role.

Dr. Harris’s serendipitous discovery of CrM efficacy, published in 1992, revolutionized creatine supplementation research [2]. Furthermore, this paper provides insights into how these discoveries shaped future research and practical guidelines, specifically carbohydrate-loading protocols for endurance and creatine strategies for performance and health. The review highlights CrM’s evolution from its original focus on sports performance to its current applications in active aging and various medical uses [3,4].

## 2. Early foundations in muscle metabolism research

### 2.1. Dr. Jonas Bergström’s development of the muscle biopsy technique

The pioneering work of Drs. Bergström and Hultman in the 1960s revolutionized our understanding of muscle metabolism, exercise physiology, and sports nutrition [5]. Their groundbreaking research on muscle glycogen content and its relationship with exercise and diet laid the foundation for modern sports science. Dr. Bergström’s research journey began during his PhD studies at the Karolinska Institute, where he initially focused on kidney disease. According to Dr. Harris, in the 1960s, repeated dialysis was suspected to cause fatigue and other medical problems in patients due to electrolyte loss, prompting Dr. Bergström to investigate this effect [6]. Recognizing that skeletal muscle is a significant reservoir of electrolytes in the human body, Dr. Bergström modified the technique described by Drs. Polley and Bickel [7] and developed the percutaneous muscle biopsy needle technique in 1959. This innovation allowed direct sampling of muscle tissue. In 1962, Dr. Bergström published his seminal paper, “Muscle electrolytes in man determined by neutron activation analysis on needle biopsy specimens” [8]. This modified biopsy technique proved instrumental in advancing our understanding of muscle metabolism during exercise. Dr. Bergström’s work laid the foundation for subsequent studies in this field, revolutionizing the approach to examining muscle energetics directly and further understanding the metabolic control of fatigue.

### 2.2. Dr. Eric Hultman’s blood glucose measurement method

At St. Eriks Hospital, Dr. Eric Hultman, an MD specializing in clinical chemistry, developed a highly specific assay for blood glucose using ortho-toluidine in glacial acetic acid. He published this technique in the journal Nature, titled Rapid, Specific Method for Determination of Aldosaccharides (simple sugars) in Body Fluids [9]. This state-of-the-art technique preceded the use of rapid reaction analyzers (i.e. glucose monitors) and set the stage for future biochemical assays.

### 2.3. Collaborative efforts linking diet and exercise performance

**Figure 1.**
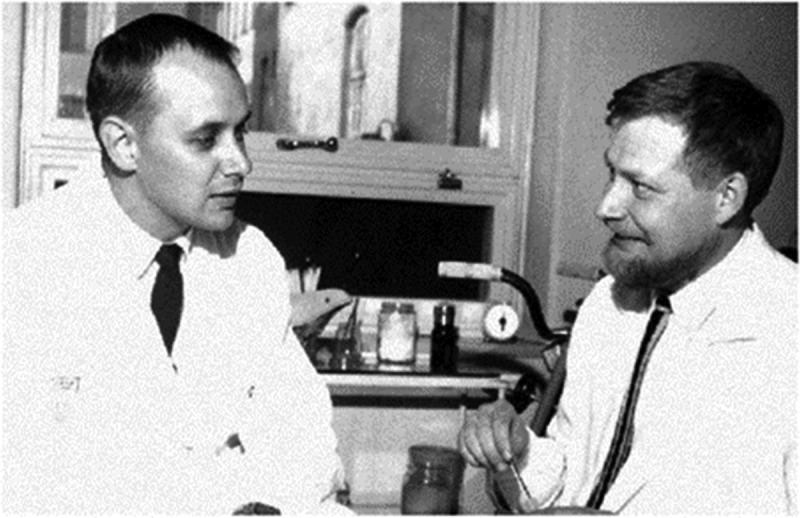
Jonas Bergström and Eric Hultman.

Drs. Bergström and Hultman, (Figure 1) who worked at the same hospital, united their expertise to tackle a groundbreaking research question. Combining Dr. Bergström’s muscle biopsy technique with Dr. Hultman’s glucose-ortho-toluidine assay, they explored the possibility of measuring glycogen in human muscle, a feat that had never been accomplished and its relationship to exercise.

These methodological breakthroughs in biopsy techniques and glucose assays established the foundation for understanding the relationship between muscle glycogen and performance. This pioneering work led directly to landmark studies that would transform our understanding of muscle glycogen’s role in endurance performance.

## 3. Landmark studies on muscle glycogen and exercise

### 3.1. 1966: quantifying human muscle glycogen

In 1966, Drs. Bergström and Hultman published their seminal study, “Muscle Glycogen Synthesis After Exercise: An Enhancing Factor Localized to Muscle Cells in Man,” in the journal Nature [1]. For the first time, this study employed Dr. Bergström’s novel muscle biopsy technique in conjunction with Dr. Hultman’s glucose-ortho-toluidine assay to quantify human muscle glycogen. The experimental design was elegant in its simplicity. Drs. Bergström and Hultman simultaneously performed unilateral cycling on opposite sides of a bicycle ergometer (i.e. Dr. Bergström exercised his left leg while Dr. Hultman exercised his right leg) until both were exhausted. Immediately after fatigue was reached, reciprocal muscle biopsies were performed in both the exercised and rested legs. Following this initial exercise session, the researchers adhered to a high-carbohydrate diet for three days. Additional muscle biopsies were performed on both legs on the morning of the second and third days [10]. Analysis of biopsy samples revealed several key findings. Immediately post-exercise, the glycogen content in the exercised leg was significantly reduced, while the glycogen levels in the resting (control) leg were unchanged. Interestingly, after just one day of a high-carbohydrate diet, the glycogen content in the exercised leg recovered and surpassed that of the rested leg. This supercompensation effect continued for the remainder of the dietary intervention, culminating in glycogen levels in the exercised leg achieving 50–60% higher stores than those of the non-exercised leg by the third day.

Practical Implications: This research laid the foundation for modern carbohydrate-loading protocols. Today, endurance athletes often increase carbohydrate intake (e.g. 7–12 g/kg body mass per day) in the days leading up to competition to maximize muscle glycogen stores, an approach that directly descends from these early findings.

### 3.2. 1967: influence of diet on endurance exercise

In a subsequent study conducted by Drs. Bergström, Saltin, and Hermansen examined the effects of high- and low-carbohydrate diets on exercise performance [11]. They discovered that a low-carbohydrate, predominantly fat diet maintained low post-exercise glycogen levels for several days, while a high-carbohydrate diet resulted in rapid resynthesis and an overshoot in glycogen content (glycogen loading). They were able to manipulate muscle glycogen levels in three states: low (glycogen depletion), medium (normal), and high (glycogen loading), before performing endurance exercise at 70% ṼO_2_max. These findings allowed them to manipulate muscle glycogen levels before exercise, revealing a near-linear correlation between exercise duration and starting glycogen content. This research marked the first time diet and nutrition had been shown to directly affect exercise performance.

While these findings established glycogen’s crucial role in endurance capacity, Drs. Bergström and Hultman recognized the need to explore broader metabolic processes. This understanding prompted investigations into the complex mechanisms regulating muscle function and fatigue.

## 4. Expansion of muscle metabolism research

### 4.1. Establishment of the Metabolic Research Laboratory

Dr. Bergström and Hultman’s success resulted in establishing a research group at St. Eriks Hospital, initially named the Bergström-Hultman Forskargrupp. According to Dr. Harris, this group, later recognized as the Metabolic Research Laboratory, expanded the muscle biopsy techniques beyond glycogen analysis. The laboratory’s primary focus was basic research in muscle energetics, aiming to extend knowledge in the metabolic control of muscle fatigue.

### 4.2. Contributions of Dr. Norman McLennan-Anderson

In 1966, Drs. Bergström and Hultman recruited a young biochemist from the United Kingdom (UK), Dr. Norman McLennan-Anderson, to investigate intermediary metabolism and the effects of exercise on adenosine triphosphate (ATP) and phosphorylcreatine (PCr) in human muscle. Their subsequent analysis of ATP and PCr level changes in muscle biopsies revealed crucial insights [12] While the initial findings suggested that human skeletal muscle might differ significantly from other mammalian species, subsequent improvements in analytical procedures later disproved this notion. A paper published in 1967 by Drs. Hultman, Bergström, and McLennan-Anderson represented a noteworthy milestone, establishing the foundation for future investigations of creatine loading and carnosine elevation [12]. This research demonstrated the critical role of high-energy phosphates in performance, foreshadowing later breakthroughs in CrM research. According to Dr. Harris, this publication stands as the most significant yet under-recognized advancement in the development of modern sports science and sports nutrition.

### 4.3. Dr. Kent Sahlin’s research on muscle pH and fatigue

Following Dr. Kent Sahlin’s arrival at the St. Eriks Metabolic Research Laboratory in 1972, research priorities evolved to examine changes in muscle pH during exercise. Prior to the development of magnetic resonance spectroscopy (MRS), researchers could only homogenize the muscle with glycolytic inhibitors and measure the pH with a microelectrode. Unfortunately, this process caused PCr degradation, elevating inorganic phosphate (Pi) and compromising the accuracy of pH measurements.

In their seminal paper, Dr. Sahlin and colleagues [13] detailed the group’s methodology for addressing this challenge, building upon earlier works by Noda et al. [14] and Siesjo et al. [15]. Their research demonstrated how declining pH negatively influences the functioning of the creatine kinase (CK) system.

The expansion of muscle metabolism research into glycogen, ATP/PCr dynamics, and pH regulation created a comprehensive foundation for understanding energy systems (Table 2). This scientific framework proved essential for Dr. Roger Harris’s subsequent breakthroughs in sports nutrition.

**Table 2.** Insights from Harris and Sahlin on Creatine in muscle function.

Key Insights
An increased ADP concentration compromises the muscle cross-bridge cycling rates, reducing muscle power and increasing fatigue. ADP spikes during muscle contractions can impair ATPase-mediated systems and reduce the energy release during ATP hydrolysis.
The primary function of the creatine/creatine kinase (Cr/CK) system is to maintain a low ADP concentration, that is, a high ATP/ADP ratio. This helps prevent significant increases in ADP levels, which could otherwise inhibit muscle contractility.
Factors that impair the function of the Cr/CK system result in increased adenine nucleotide (AN) degradation and the early onset of fatigue. The CK reaction is crucial for buffering ADP spikes via PCr-mediated phosphotransfer, which is essential for maintaining muscle function.
Although several guanidino phosphagens perform similar functions, the Cr/CK system is the most effective at maintaining a high ATP/ADP ratio. However, it is also the most unstable, with a loss of 1.74% daily. The rapid resynthesis of PCr after exercise, which is dependent on ATP from oxidative phosphorylation, highlights the importance of the system and its role in muscle recovery and oxidative potential.

## 5. Dr. Roger Harris’s pivotal role in sports nutrition

### 5.1. Initial contributions at the Metabolic Research Laboratory

Dr. Roger Harris joined the Bergström-Hultman Forskargrupp at the end of 1968, overlapping briefly with Dr. Norman McLennan-Anderson. Harris’s first task was to validate, rationalize, simplify, and extend the analysis employed in the 1967 paper on phosphagen changes with exercise. This work used the fundamental texts of HU Bergmeyer [16] and Passonneau and Lowry [17]. Dr. Harris’s work resulted in the publication of a highly influential paper in 1974, which provided reference data for ATP, PCr, creatine, and total creatine (TCr) that served as the basis for subsequent MRS studies for many years. This article, titled “Glycogen, glycolytic intermediates, and high energy phosphates were determined from biopsy samples from the musculus quadriceps femoris of a man at rest,” has been cited more than 1500 times [18].

### 5.2. Challenges and insights in early creatine research

In 1975, the group attempted to increase muscle creatine levels with CrM. However, a preliminary study involving two participants (both swimmers with elevated baseline creatine levels) showed minimal improvement, and the laboratory incorrectly concluded that CrM supplementation could not significantly raise muscle creatine levels. Due to skepticism stemming from this unpublished pilot study, research into creatine essentially halted for nearly 15 years.

Although initial skepticism about creatine supplementation temporarily halted progress, Dr. Harris’s transition to equine muscle metabolism research unexpectedly catalyzed discoveries that would revolutionize the field.

## 6. Transition to equine research and serendipitous discoveries

### 6.1. Dr. Harris’s work at the Animal Health Trust

In 1983, Dr. Harris joined the Animal Health Trust in Newmarket, UK, where he continued his research on muscle metabolism, now focusing on horses. Although his primary interest shifted to equine physiology, these studies indirectly reignited Harris’s curiosity about creatine supplementation in humans. During his time at the Animal Health Trust, Dr. Harris established connections with Lonza, a company involved in carnitine production. These discussions about various muscle metabolites, including carnitine, carnosine, and creatine, were crucial in advancing creatine supplementation for human use.

### 6.2. The accidental path to creatine supplementation

#### 6.2.1. The story of Sammy the Racehorse

**Figure 2.**
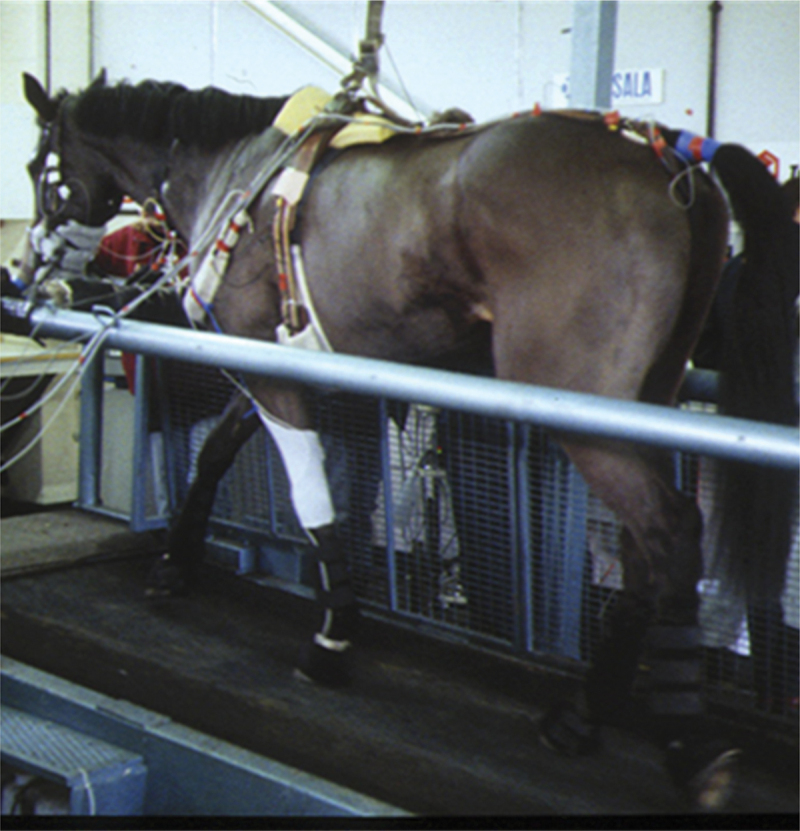
Sammy the Racehorse (courtesy of Roger Harris).

A breakthrough in creatine supplementation occurred somewhat serendipitously when Dr. Harris received a sample of CrM instead of carnosine from Lonza. Initially, Dr. Harris left the 50 g sample on the laboratory bench for several months, as he had not requested it and did not want it due to a previous unsuccessful attempt in 1975. Eventually, Dr. Harris decided to experiment on a racehorse named Sammy (Figure 2) to test this supplement.

On a late October morning, Dr. Harris planned to investigate the oral administration of CrM to Sammy the Racehorse. The protocol involved dissolving CrM in water and administering it orally via a 50 ml syringe, followed by the collection of blood samples for two hours. However, Dr. Harris encountered challenges due to the unexpectedly low solubility of CrM in water. The attempt to dissolve 10 g in 50 ml of water proved problematic, with a significant portion of the substance remaining in suspension, even after an extended period. After a prolonged and unsuccessful attempt to prepare the solution, Dr. Harris administered the creatine anyway. However, the crystalline nature of the undissolved CrM caused the syringe plunger to seize, ultimately forcing the abandonment of the equine trial.

##### 6.2.1.1. Self-experimentation leading to the human breakthrough

Despite this equine setback, Dr. Harris pivoted to self-experimentation. After consulting the solubility data, 5 g of CrM was measured and dissolved in 300 ml of warm water. Dr. Harris then ingested this solution and ordered blood samples to be taken 30 and 60 minutes after ingestion, finding a notable rise in plasma creatine, which directly contradicted the group’s 1975 conclusions. This unexpected finding prompted immediate collaboration with Dr. Hultman to investigate CrM absorption and muscle uptake in humans.

These serendipitous findings prompted Harris and colleagues to reexamine creatine supplementation in humans, leading to their groundbreaking 1992 discovery that revolutionized sports nutrition.

## 7. The breakthrough in creatine supplementation (1992)

### 7.1. Human study demonstrating effectiveness

After self-experimentation, Dr. Harris initiated a human study at the Huddinge University Hospital in Stockholm. This study was conducted in collaboration with Drs. Hultman and Karin Soderlund. Dr. Soderlund performed most of the analysis, Dr. Hultman performed the biopsies, and Dr. Harris organized the data, wrote the paper, and drew the graphs. The 1992 publication in Clinical Science, initially rejected by Nature and the Journal of Physiology, changed the landscape of modern sports nutrition by clearly demonstrating that oral CrM supplementation elevated muscle creatine stores [2].

### 7.2. Key findings and physiological implications

Harris et al. (1992) conducted a study that involved 17 subjects (5 females and 12 males) aged 20–62 years, including two vegetarians. Subjects received 5 g doses of CrM four to six times daily for periods ranging from 4.5 to 21 days. A 5 g dose increased plasma creatine concentrations to a mean peak of 795 μmol/l after 1 h, equivalent to a creatine content of 1.1 kg of fresh uncooked steak. Further results showed that CrM significantly increased the TCr content in the quadriceps femoris muscle, with the highest absolute increase observed in subjects with the lowest initial levels of TCr. The mean TCr content increased from 126.8 mmol/kg dry muscle (DM) before supplementation to 148.6 mmol/kg DM after. In particular, exercise with one leg combined with supplementation led to even more significant increases in the exercised leg (up to 182.8 mmol/kg DM in one subject). The study also found no adverse effects from CrM, with optimal results achieved through repeated doses of 5 g, leading to significant increases in muscle creatine and PCr levels without affecting the ATP content. The study also noted that 20–40% of the increase in TCr was PCr. These findings laid the groundwork for the widespread acceptance of CrM as an evidence-based ergogenic aid and fueled subsequent research that expanded into additional applications such as active aging, sarcopenia, and clinical populations [4].

The validation of CrM’s effectiveness sparked rapid adoption among elite athletes and launched a new era in sports nutrition. This breakthrough’s broader implications and enduring legacy continue to influence both athletic performance and therapeutic applications.

## 8. Real-world impact and legacy

### 8.1. Adoption by athletes and influence on performance

#### 8.1.1. The Barcelona Olympics and Ergomax

**Figure 3.**
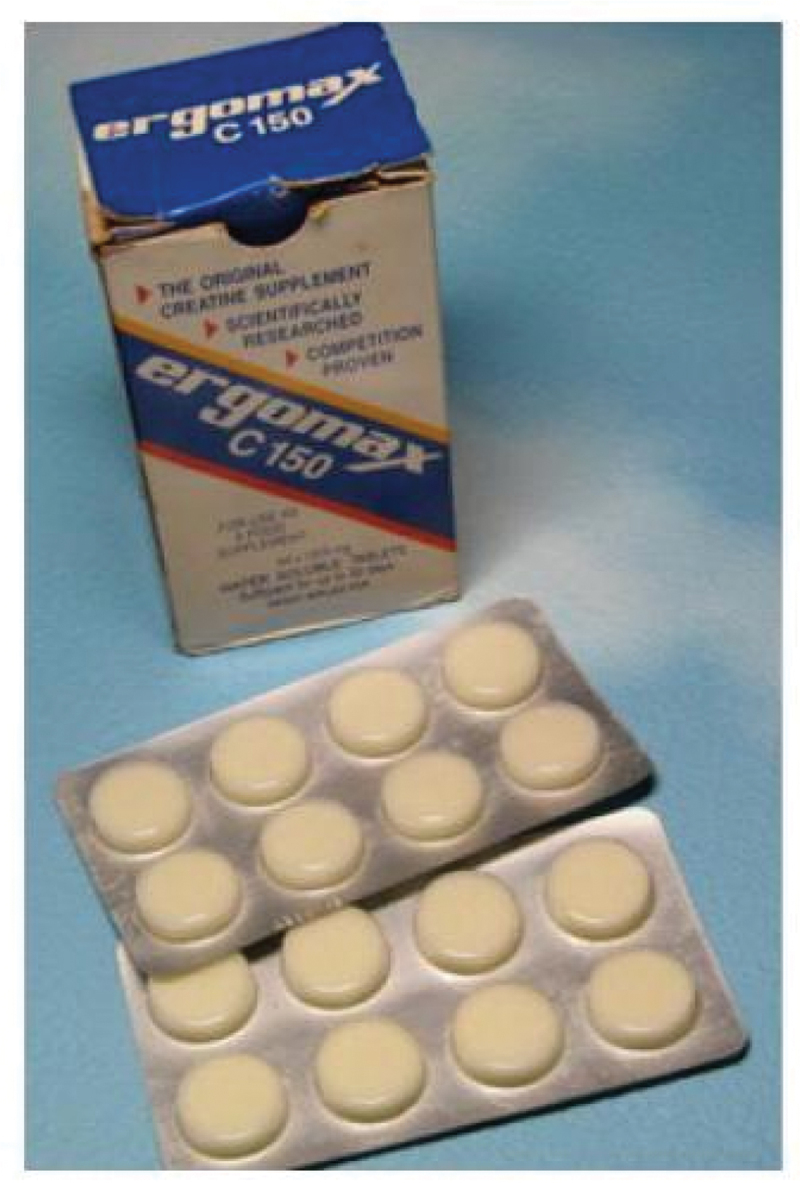
Ergomax – first creatine monohydrate product for athletes (courtesy of Roger Harris).

The publications on creatine loading by Harris et al. [2] and Hultman et al. [19] had a profound impact on sports nutrition, igniting widespread interest in CrM among athletes and researchers. A 1992 newspaper article by Doug Gillon [20] in *The Herald Scotland* described how scientists, including Dr. Harris, developed Ergomax (Figure 3), a CrM product reportedly used by over 100 British athletes during the Barcelona Olympics. High-profile athletes like Colin Jackson and Linford Christie claimed it helped boost their performances.

### 8.2. What if the administration of creatine monohydrate to Sammy had been successful?

Some years later, Dr. Harris supervised his PhD student investigating creatine supplementation in horses. The study found only a modest increase in plasma creatine levels after a single dose. Additionally, following chronic dosing of creatine (~50 g per day), there was no significant change in muscle creatine concentration [21]. This finding was confirmed in two studies by Dr. Birgitta Essen-Gustavsson’s research group, which showed no change in plasma creatine or muscle TCr after supplementation with creatine in a daily dose of 50 g^22,23^. These results contradict the Harris et al. [2] study in humans, which showed that creatine supplementation significantly increased plasma creatine. This suggests differences in the absorption mechanism between species, with horses being herbivores, having delayed or diminished creatine absorption compared with humans [21–23]. Dr. Harris noted that, based on the aforementioned research, they would have observed no effect if he had successfully administered the creatine solution to Sammy the horse. This outcome would have supported their belief from 1975 that creatine was not absorbed, potentially further delaying the discovery of creatine loading in humans.

It should be noted that while Drs. Chanutin and Guy showed in 1926 that creatine administered orally was initially retained, they did not identify its role in increasing muscle levels or recognize its importance in cellular energetics. The groundbreaking work by Harris et al. and Hultman et al. in the 1990s was crucial in establishing the importance of creatine in sports nutrition and performance improvement.

### 8.3. Broader implications for ergogenic aids and sport nutrition

Identifying the ergogenic effects of CrM in humans sparked exploration into other supplements, such as beta-alanine (a precursor for carnosine synthesis in skeletal muscle). This interest in buffering capacity had its roots in the Metabolic Research Laboratory in the mid-1970s. Over the last few decades, sports nutrition has expanded from focusing primarily on carbohydrate intake for endurance to a robust field encompassing protein supplementation, beta-alanine [24], and other dietary strategies to enhance performance at all levels. There are now peer-reviewed journals dedicated to sports nutrition, including the International Journal of Sports Nutrition (now known as the International Journal of Sport Nutrition, Exercise, and Metabolism [IJSNEM]) and the Journal of the International Society of Sports Nutrition (JISSN). In addition, there are now professional organizations and certifications (e.g. the International Society of Sports Nutrition and the Collegiate and Professional Sports Dietitians Association), further solidifying the field’s credibility.

Ironically, there was initial resistance and a lack of support for sports nutrition; however, there is evidence that sports nutrition research is accepted and credible and has influenced other health-related research fields, such as age-related health research. For example, several dietary interventions used to enhance “performance” in athletes (e.g. creatine, protein) may improve “function” in older adults. Importantly, this paper is not meant to be a comprehensive examination of all sports nutrition research; its purpose is to highlight the Metabolic Research Laboratory and a few key moments that significantly impacted the field.

The far-reaching impact of CrM research exemplifies how these pioneers’ methodical investigations and serendipitous discoveries transformed sports nutrition. Their foundational work continues to influence modern scientific practices and athletic performance.

## 10. Conclusion

Dr. Bergström’s muscle biopsy technique, introduced in 1966, represented a fundamental shift in muscle physiology and sports nutrition research, transforming the field from empirical methods to evidence-based interventions. By facilitating direct assessment of muscle glycogen and other metabolites, this method established definitive evidence of how diet influences exercise performance.

The significance of this methodological advancement is demonstrated by the thousands of studies published since its introduction, with PubMed searches documenting over 2,000 papers on skeletal muscle metabolism using biopsy techniques, more than 1,000 on muscle energetics, and over 500 on fiber typing.

Building on this foundation, Drs. Bergström and Hultman demonstrated the direct impact of diet on muscle glycogen levels and exercise performance, establishing core principles of modern sports nutrition. The subsequent establishment of the Metabolic Research Laboratory, with contributions from researchers such as Drs. Norman McLennan-Anderson, Kent Sahlin, and Roger Harris, deepened our understanding of muscle energetics and fatigue.

A pivotal development came through Dr. Harris’s reexamination of creatine supplementation, revealing that oral creatine intake could significantly increase intramuscular creatine content and enhance performance. This breakthrough transformed the practice of sports nutrition, leading to the widespread adoption of CrM by athletes and researchers.

The significant advances made in the 20th century, ranging from Dr. Bergström’s biopsy techniques to Dr. Robert Cade’s creation of Gatorade, have revolutionized our understanding of how macronutrient intake and hydration affect exercise performance and recovery. As sports nutrition continues to evolve, new supplements and strategies emerge, but the foundational studies by Drs. Bergström, Hultman, and Harris remain a cornerstone. Their pioneering contributions continue to guide future research, underscoring the lasting importance of their work in advancing human performance and nutrition science.

### 10.1. In memoriam

**Figure 4.**
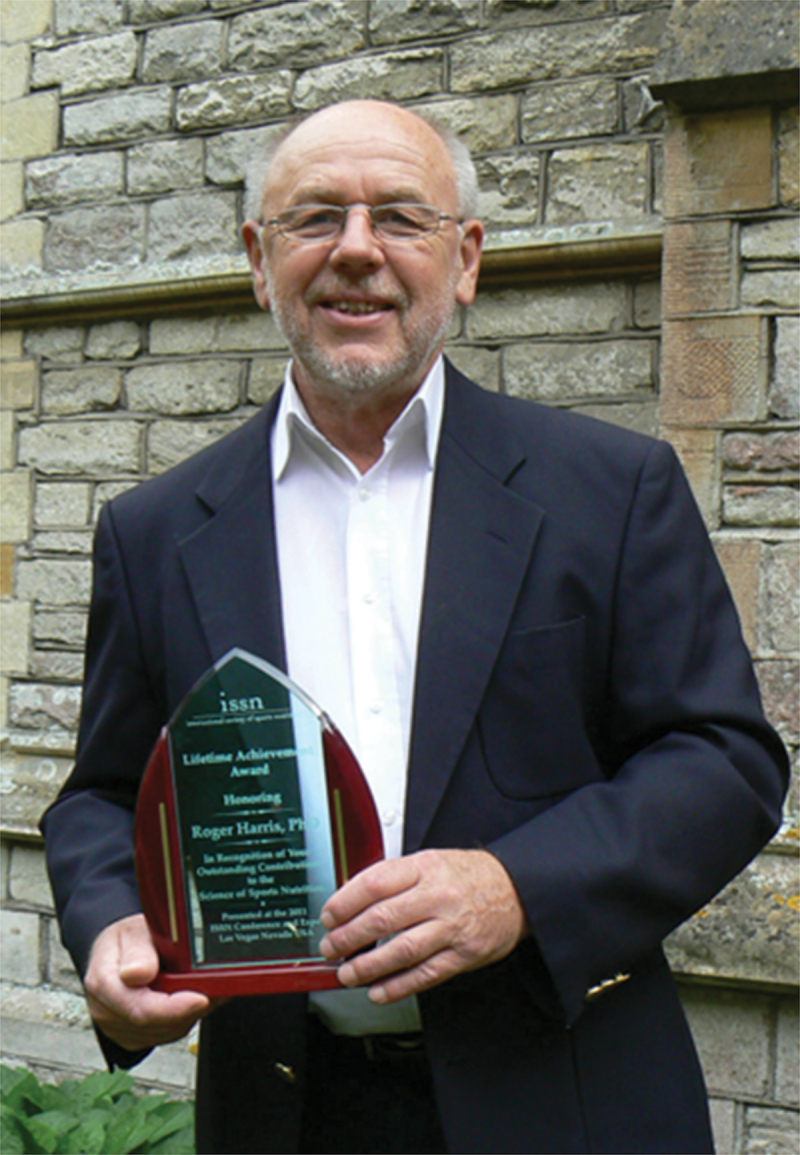
Professor Roger Harris holding the ISSN lifetime achievement Award.

The scientific community mourns the loss of Dr. Roger Harris, Ph.D., FISSN, (Figure 4) who passed away on 1 December 2024, at the age of 80. As a pioneering biochemist whose groundbreaking research fundamentally transformed sports nutrition, Dr. Harris’s legacy began in 1968 when he joined Drs. Eric Hultman and Jonas Bergström at St. Eriks Hospital in Stockholm. His work there helped transition the field from empirical observations to laboratory-based science, establishing the foundation of modern sports nutrition. His most significant contributions came through two landmark discoveries: creatine loading in human muscle tissue in 1992, and later, the elevation of muscle carnosine through beta-alanine supplementation. These findings revolutionized athletic performance nutrition and continue to influence Olympic athletes and sports science today.

Throughout his distinguished career, which included serving as Chair of Exercise Biochemistry and Sports Science at the University of Chichester until his retirement in 2009, Dr. Harris authored over 200 research papers and numerous book chapters. His dedication to advancing exercise biochemistry earned him the International Society of Sports Nutrition’s first-ever Lifetime Achievement Award in 2011. Even in retirement, his passion for research continued through collaborations with institutions worldwide, including the Korean National Sports University, the University of São Paulo, the University of Central Florida, and Nottingham Trent University. Dr. Harris’s humility and collaborative spirit were evident in his frequent acknowledgment that his achievements represented “the work of many collaborators and research students.” His pioneering work has left an indelible mark on sports nutrition and exercise science that will benefit athletes and researchers for future generations.
